# The Effectiveness of Compassion-focused Therapy on Patients with Bipolar Disorder, A Randomized Clinical Trial

**DOI:** 10.31661/gmj.v13i.3359

**Published:** 2024-09-11

**Authors:** Forouzan Behrouzian, Hatam Boostani, Esmaeil Mousavi Asl, Neda Sadrizadeh, Shima Nemati Jole Karan

**Affiliations:** ^1^ Department of Psychiatry, Golestan Hospital, School of Medicine, Ahvaz Jundishapur University of Medical Sciences, Ahvaz, Iran

**Keywords:** Compassion-focused, Psychotherapy, Clinical Trial, Therapy, Bipolar Disorder

## Abstract

Background: Multifarious complications are attributed to the bipolar disorder; while existing treatments are not yet fully effective. In this study we evaluated effects of Compassion-focused therapy (CFT), an integrated psychological technique to include kinder thinking habit.Materials and Methods: This was a randomized trial (IRCT20211206053298N1) on a total of 26 individuals satisfying the diagnostic criteria for bipolar disorder who were allocated in two study arms by simple random allocation method, first receiving both CFT and conventional therapy (n=13) and seconds receiving traditional therapy alone (n=13). Compassion-focused therapy received consisted of ten sessions of CFT in conjunction with the customary treatment. Various psychological constructs, including self-compassion, self-criticism, shame, and distress associated with bipolar disorder were examined at beginning of study, after finishing the sessions, and 2 months later. Data analysis involved using repeated measures analysis of variance (RM-ANOVA) with statistically significant threshold of under 0.05 in SPSS V. 21.Results: There were no differences in educational level, gender, occupation, history of hospitalization, medical illness, and marital status between the study groups (P0.05). There was a significant difference in trend of changes of studied variables. Intervention group showed a significant sharp decrease in shame (from baseline value of 12.76±4.38 to 4.46±7.26 two months later), self-criticism scores (26.61±6.57 at baseline and 14.76±5.74 at two-months period), and distress scores (21.0±2.29 at baseline to 11.75 ±2.00 at two-months period) from study beginning to final follow up that also had a more substantial decline than the control group (P0.05). Both groups showed increased self-compassion score during the time with no differences among their increasing trend (P=0.725).Conclusion: The effect of CFT on the psychological treatment outcomes for individuals diagnosed with bipolar disorder appears to be significant, while it was not as effective on the self-compassion score. So, further studies would be helpful to draw a more precise conclusion.

## Introduction

Bipolar disorder, categorized as a mood disorder, is characterized by pronounced shifts in mood [1]. Typically, the disease begins with a phase of depression, followed by the emergence of manic episodes after one or more depressive episodes [2]. However, in a smaller subset of patients, the onset of the condition may be characterized by manic or hypomanic episodes [3]. The most current edition of the Diagnostic and Statistical Manual of Mental Disorders (DSM-5) recognizes bipolar disorder as a distinct and separate condition [4]. According to the DSM-5 report, the estimated 12-month prevalence of bipolar disorder type 1 (BP-I) in the United States stood at approximately 0.6% [4]. The lifetime prevalence of BP-II is approximately 1.1% in US [5]. Furthermore, in Iran, there is a reported prevalence of 29.4% for mood disorders, with bipolar disorder specifically having a prevalence rate of 0.96% [6]. In the Global Burden of Diseases report by the World Health Organization, bipolar disorder has been recognized as the sixth most incapacitating condition worldwide [7]. In the age range of 15 to 44 years, bipolar disorder is ranked as the fifth highest contributor to disability [8]. Bipolar disorder stands as a profoundly severe mental health issue with significant long-term consequences [9]. Psychologists emphasize that the effect of biological and social factors needs to be examined through the prism of an individual’s psychological inclinations. Among these psychological variables, shame stands out as a noteworthy aspect [10]. One of the negative emotions, shame, encompasses the perception that others view an individual in an unfavorable light, perceiving them as being of low worth, inadequate, undesirable, or unappealing [11]. The findings of meta-analyses demonstrated a correlation between shame and self-harming behaviors, including non-suicidal self-injury [12][13]. Bipolar disorder is also connected to the presence of self-critical tendencies. Self-criticism pertains to the maladaptive regulation of emotions, manifesting as a punitive and adverse internal dynamic wherein one evaluates themselves harshly in response to errors, mistakes, or qualities that could lead to rejection or negative social judgment [14][15][16]. While medication therapy is commonly regarded as the primary approach for managing the condition, there have been reports of an efficacy-effectiveness gap in terms of the response rate to all mood stabilizers [17]. Despite the considerable advantages of drug therapy, approximately 40% of individuals with bipolar disorder experience a relapse within the initial year [18]. Conversely, the current psychological interventions exhibited low or deficient quality [19]. Given the limitations associated with current pharmacological and psychological therapies, novel psychological interventions has emerged as well as the Compassion-Focused Therapy (CFT). Assisting individuals with bipolar disorder in cultivating an inner disposition characterized by warmth, love, care, and compassion toward their personal experiences stands as a pivotal aspect of CFT. [20]. Rosenfarb et al. [21] demonstrated that the implementation of CFT yielded positive outcomes concerning body image and marital satisfaction among women diagnosed with breast cancer. As shame and self-criticism exhibit a strong connection with bipolar disorder, and taking into account the constraints of current pharmacological and cognitive behavioral therapy approaches, this study aimed to examine the efficacy of CFT in addressing the outcomes of individuals undergoing treatment for bipolar disorder (both BP-I and BP-II).

## Materials and Methods

Study Design

This study employed a randomized clinical trial (RCT) utilizing a pre-test-post-test and follow-up design involving two groups. It included individuals diagnosed with bipolar disorder who sought treatment at the psychiatric clinic of Golestan Hospital, Jundishapur University of Medical Sciences, Ahvaz, from 2001 to 2001.

Study Population

Inclusion criteria for the study were individuals aged 18 to 50 years, meeting the diagnostic criteria for bipolar disorder, for both types of BP-I and BP-II as outlined by SCID-5-CV, absence of an acute phase of the illness, and adherence to a consistent medication regimen. Conversely, individuals were excluded from the study if they presented with brain damage, dementia, or specific neurological disorders, had not received electroconvulsive therapy during the treatment period, had severe physical ailments such as cancer, or were concurrently engaged in other psychotherapeutic interventions utilizing the same measurement tools.

Sampling Method, Sample Size Determination, and Randomization Method

A total of 47 patients were selected by implementing a convenience sampling method and subsequently allocated randomly into two separate groups. Random allocation was performed by assigning a unique code to each participant and utilizing a computer to generate a random sequence of numbers. Our study sample size was powered based on the self-compassion assessments of Gharraee et al. [22] who performed a similar RCT on CFT for anxiety disorder; with a large Cohen’s d effect size detected (d>0.8), and employing a desired significance level of 0.05 and power of 0.8, a minimum sample size of 12 participants per group has been established for the current investigation.

Study Arms

The study included two groups: the first group received a combination of compassion-focused therapy and conventional therapy, while the second group solely received traditional therapy. The structure and content of the CFT sessions were developed concerning the treatment approach formulated by Paul Gilbert (1) and Russell Colts (2). The treatment protocol was applied per the treatment session content, with selective utilization of specific techniques such as exposure therapy, compassionate imagery, soothing breathing exercises, and mindfulness practices tailored to the client’s needs. Before commencing each session, a comprehensive review was conducted, encompassing the previously discussed topics and the completion of any assigned home exercises. The primary focus of the session revolved around instructing and elucidating therapeutic concepts. Ultimately, the treatment session concluded by providing a summary, establishing the objectives for the upcoming session in collaboration with the relevant individuals, and acquiring feedback.

Outcome Measures and Follow ups

Four primary variables were investigated: shame, self-compassion, self-criticism, and distress associated with bipolar disorder. For this study, the assessment tools for diagnosing BP included the structured clinical interview tools SCID-V-CV. The Structured Clinical Interview for DSM-IV Axis I Disorders, Version 5 (SCID-V-CV) is a widely accepted semi-structured clinician-administered diagnostic instrument developed to facilitate accurate psychiatric diagnoses based on the Diagnostic and Statistical Manual of Mental Disorders, Fourth Edition (DSM-IV) criteria (1).

-The Distress Scale Related to Bipolar Disorder - short form (DISBIP-S) is a brief instrument designed to quantify subjectively experienced emotional distress linked to bipolar disorder. We used psychological/cognitive distress dimension of the questionnaire with questions of:

Q1: How often do you feel sad, angry, and/or scared when thinking about living with a bipolar disorder?

Q2: Do you believe your bipolar disorder influences your personality?

Q3: Do you perceive your mental abilities are negatively affected by your bipolar disorder?

Q4: Do people with bipolar disorder carry a heavy burden because of the illness?

Q5: Does having bipolar disorder cause you considerable distress that you struggle to manage?

Based on a 1 to 10 Likert scale, the total score of this questionnaire was summed from mentioned 5 questions. A higher score shows higher distress [23].

-The Short Form of the Self-Compassion Scale (SFSCS), a comprehensive 18-item measure specifically designed to evaluate self-compassion, was also utilized. The scoring system for this scale employs a five-point Likert scale, ranging from "never" to "always," and demonstrates favorable psychometric characteristics. Six core components of self-compassion include: Self-Kindness, Self-Judgment, Common Humanity, Isolation, Mindfulness, and Overidentification. Sample questions include "I try to be gentle and understanding with myself when I make mistakes" (Self-Kindness); "I can be extremely hard on myself when I mess up" (Self-Judgment); "When I suffer, I remember that I am not alone" (Common Humanity); "When I feel inadequate in some way, I tend to isolate myself" (Isolation); "When I’ve made a big mistake, I try to observe my feelings without getting caught up in them" (Mindfulness); and "When I feel bad about myself, I tend to obsess and fixate on everything that’s wrong" (Overidentification). Additional items address acceptance vs. rejection and connections vs. disconnection. [24].

The Self-Critical Rumination Scale (SCRS) is a ten-item questionnaire designed to assess the extent of self-critical rumination. Participants provide their responses to these items using a four-point scale, ranging from "not at all" (1) to "completely good" or "very good" (4), with example items like "After a disappointment or setback, I spend a lot of time reviewing what went wrong in my mind" and "When I think about past events, I tend to criticize myself for the mistakes I made". This scale demonstrates favorable psychometric properties [25].

The shame scale comprises eight items and serves as a tool for assessing both internal and external aspects of shame. Participants rate each item on a five-point Likert scale, ranging from "never" (0) to "always" (4), exemplified by "When I make a mistake, I feel terrible and embarrassed" and "Other people look down on me when I admit a mistake.". This scale demonstrates favorable psychometric properties [26]. The dependent variables, including self-criticism, bipolar disorder-related anxiety, shame, and self-compassion, were assessed at three distinct time points: before treatment initiation, after treatment completion, and two months following the conclusion of treatment (follow-up). The CFT spanned ten sessions, with each session occurring every week. Questionnaires were administered before the start of treatment, immediately following the final treatment session, and two months after the conclusion of the last treatment session. The therapy sessions were one-on-one, involving the therapist and the patient. The participants attended one session per week, and the questionnaire was completed before the initial session, immediately following the final session, and two months after the last session. The researcher and therapist involved in this study were skilled psychiatric residents with comprehensive training in compassion-focused therapy. This training encompassed theoretical and practical aspects, was overseen by experienced supervisors and consultants, and included ongoing clinical supervision. To ensure treatment integration and adherence to the research protocol, the therapy sessions were conducted with the guidance and oversight of supervisors and counselors. This supervision assessed the therapist’s loyalty to the relevant principles and guidelines. No one was blinded to interventions; while statistician was not aware of the exact group of the data analyzing.

Data Analysis

To analyze the data, descriptive statistics were employed to calculate indices such as frequency, mean, and standard deviation, offering informative descriptions. In the inferential statistics section, the analysis of variance with repeated measures MANOVA, independent sample t-test, and chi-square test were conducted using SPSS version 23 software (IBM SPSS Statistics for Windows, version XX (IBM Corp., Armonk, N.Y., USA)).

Ethical Considerations

The participants were informed that their involvement in the study was part of a research project. To carry out the interventions in this study, ethical approval (IR.AJUMS.HGOLESTAN.REC.1400.120) was obtained from Ahvaz Jundishapur University of Medical Sciences (AJUMS). Also, the study’s protocol was registered in Iranian RCT repository (IRCT20211206053298N1).

## Results

**Figure-1:**
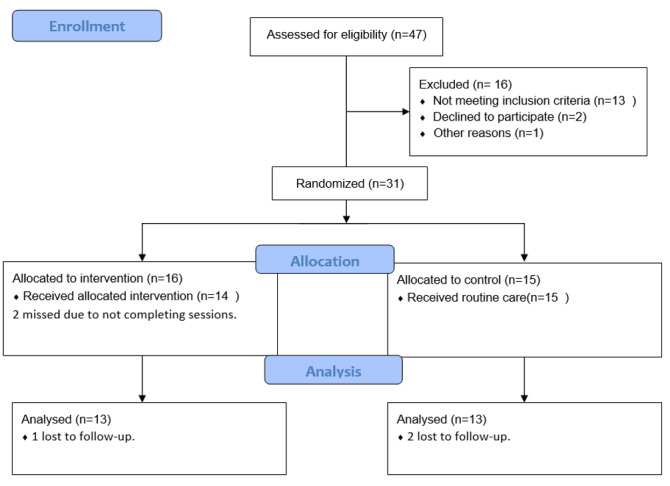
Figure 1. CONSORT Flow Diagram

**Figure-2:**
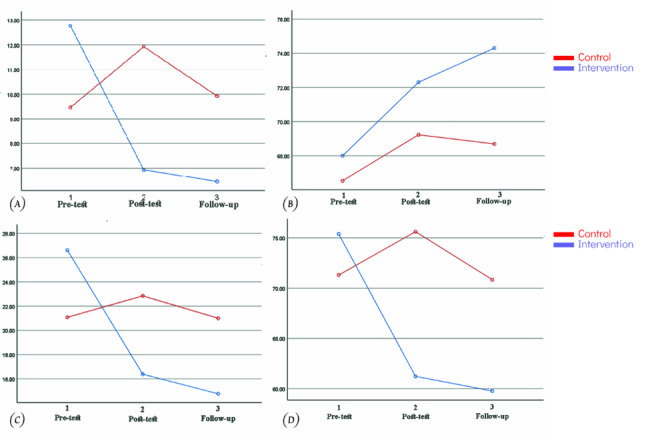
Figure 2. Comparative Analysis of Variation of Study Measures Across Three Evaluation Phases in Two Experimental and Control Groups. A: Shame score. B: self-compassion scores. C: self-criticism scores. D: distress.

**Table:** Table1. Characteristics of Included Subjects

		intervention group		control group		
n/mean		%/SD	n/mean	%/SD	mean	p
Age, mean, SD		29.15	6.44	28.46	5.86	0.77
Gender, n, %	female	11	84.62	10	76.92	0.619
	male	2	15.38	3	23.08	
	High school	1	7.69	1	7.69	
	Diploma	6	46.15	7	53.85	
Educational status, n, %	Associate Degree	2	15.38	1	7.69	0.817
	Bachelor's degree	3	23.08	4	30.77	
	Master's degree	1	7.69	0	0	
	Single	8	61.54	8	61.54	
Marital status, n, %	married	4	30.77	4	30.77	0.999
	divorced	1	7.69	1	7.69	
	employed	4	30.77	4	30.77	
Occupational status, n, %	unemployed	9	69.23	9	69.23	0.999
	housekeeper	0	0	0	0	
	1	1	7.69	1	7.69	
number of medications, n, %	2	8	61.54	5	38.46	0.639
	3	3	23.08	6	46.15	
	4	1	7.69	1	7.69	
Hospitalization counts, mean, SD		3.92	4.9	1.46	1.12	0.091

In this study, both intervention and control groups consisted of 13 individuals (as shown in Figure-1). In the CONSORT Flow Diagram (Figure-1), a total of 47 participants were initially assessed for eligibility. Of these, 16 were excluded, with 13 not meeting the inclusion criteria, 2 declining to participate, and 1 for other reasons. Subsequently, 16 participants were allocated to the intervention group, of which 14 received the allocated intervention, while 2 missed sessions. Additionally, 15 participants were allocated to the control group and all received routine care. Upon analysis, 13 participants from both the intervention and control groups were included, with 1 participant lost to follow-up in the intervention group and 2 lost to follow-up in the control group.

Of the 26 research participants, 21 (80.8%) were women and 5 (19.2%) were men. Of the 26 individuals, 25 people (96.2%) had previously experienced hospitalization, whereas only a single person (3.8%) had a medical illness. The chi-square test was utilized to assess the significance of group differences across variables such as education, gender, occupation, history of hospitalization, medical illness, and marital status. The findings revealed no notable distinction between the groups in these aspects, as shown in Table-1. Table-2 presents descriptive data on the variables of shame, self-compassion, self-criticism, and distress associated with bipolar disorder. The information is categorized into two groups and delivered across the pre-test, post-test, and follow-up stages. Furthermore as shown in Table-2 and Figure-2, the research revealed a significant effect of the measurement time on shame scores (F=3.843, P=0.028; Figure-2.A) shows the alterations observed across three evaluation stages between the two examined groups. According to the Figure-1, the intervention group displayed a reduction in shame scores during the post-test and follow-up phases (Figure-2.A). Meanwhile, the control group showed an uptick in shame scores during the post-test interval, subsequently reverting to lower figures during the follow-up phase (Figure-2.A).

Our results indicate that the point in time when measurements were taken had a significant impact on participants’ levels of self-compassion (F=3.382, P=0.042). However, further analysis showed that the relationship between time and whether or not someone belonged to the intervention or control group did not achieve statistical significance (P=0.490, F=0.725; Figure-2.B). This suggests that there is no significant variation in the mean self-compassion scores between the two groups as they are measured over different time intervals.

The results of our analysis show that the timing of measurements has a statistically significant influence on participants’ self-criticism scores (P=0.001, F=9.477), meaning that there is a notable difference in the average self-criticism scores across all three testing periods - before the intervention, after the intervention, and at follow-up. Moreover, we found a significant interaction effect between time and treatment group (F=11.862, P=0.001; Figure-2.C), which indicates that the change in self-criticism scores over time varied significantly depending on whether an individual was part of the intervention or control group.

The interaction effect between time and treatment group was statistically significant for distress scores (F=3.024, P=0.048; Figure-2. D). Subsequently, we performed pairwise comparisons to examine the differences among the various testing stages. Our analysis revealed that the discrepancies in distress scores between the pre-intervention and post-intervention assessments (P=0.034) and those between the pre-intervention and follow-up evaluations (P=0.002) were statistically significant. Conversely, no meaningful difference was detected in distress scores between the post-intervention and follow-up periods (P=1.000) (Table-3).

## Discussion

**Table:** Table2. Descriptive Data Gathered Across Research Stages for Intervention and Control Groups

Variables	Group	Pre-test Mean (SD)	Post-test Mean (SD)	Two months later Mean (SD)
Shame	Intervention	12.76 (4.38)	6.92 (6.14)	4.46 (7.26)
	Control	9.46 (8.22)	11.92 (9.98)	9.92 (9.06)
Self-compassion	Intervention	68 (8.84)	72.3 (8.37)	74.30 (10.2)
	Control	66.53 (7.06)	69.23 (6.68)	68.69 (7.44)
self-criticism	Intervention	26.61 (6.57)	16.38 (6.21)	14.76 (5.74)
	Control	21.07 (8.41)	22.83 (10.64)	21 (9.07)
Distress associated with bipolar disorder	Intervention	21 (2.29)	10.58 (2.23)	11.75 (2)
	Control	21.61 (1.75)	8.61 (2.02)	9.15 (3.41)

**Table:** Table3. MANOVA Analysis of Study Measures for Intervention and Control Groups during the Time

Variables	Within Group (Time)	Between Groups (Group × Time)
Shame	F=3.843, P=0.028	F=0.123, P=0.725
Self-Compassion	F=3.382, P=0.042	F=0.546, P=0.581
Self-Criticism	F=9.477, P<0.001	F=11.862, P=0.001
Distress	F=3.024, P=0.048	F=2.321, P=0.129

Previous studies have indicated a connection between self-criticism and depressive symptoms, both in clinical and non-clinical populations, whereby self-criticism is associated with more pronounced and severe manifestations of depression [15]. McMurrichet et al. [16] conducted a study revealing that self-criticism in family members of individuals with bipolar disorder can be predicted by factors such as talent for experiencing shame, talent for experiencing guilt, and symptoms of depression.

Bipolar disorder arises due to significant disruptions within the emotional systems and causes a considerable challenge to mental health. CFT emerged due to a neuroscientific and developmental perspective, with a specific focus on regulating emotional systems. As a consequence, it offers potential significant interventions for the treatment of bipolar disorders [27]. CFT is an approach to addressing mental health issues and providing treatment that integrates evolutionary consciousness and a biopsychosocial perspective [28]. Fundamental drives shaped by evolution, such as motivations related to care, cooperation, and competition, serve as primary sources of organizing psychophysiological processes that underlie mental health issues [28]. Consequently, psychotherapy can focus on addressing evolved motivations as focal points for intervention. According to the findings of Gilbert et al. [28], participants demonstrated the ability to transition from a mindset centered around competition to one centered around compassion, leading to subsequent improvements in mental well-being and social behavior. Throughout the study, participants underwent a progression from a cognitively-based comprehension of cultivating a compassionate mindset to a more experiential and embodied sense of compassion, which had notable impacts on their approach to the psychological aspects of bipolar disorder [28].

McManus et al. [29] provided evidence to support the viability and acceptability of integrating CFT into the care provided by community mental health teams for clients across various psychiatric diagnoses. The research conducted by Palmer-Cooper et al. [30] revealed that self-compassion serves as a partial mediator in the connection between negative cognitions and mood outcomes in individuals diagnosed with bipolar disorder. The research conducted by Palmer-Cooper et al. [30] revealed that self-compassion serves as a partial mediator in the association of negative cognitions and mood outcomes in individuals diagnosed with bipolar disorder. The primary objective of implementing CFT in individuals diagnosed with bipolar disorder is to foster the development of a self-optimized relationship rooted in a mindset centered around care and soothing. Within this framework, individuals are instructed to monitor their thoughts, evaluate their nature, document thoughts and emotions linked to self-disappointment and self-criticism, and subsequently shift their attention toward cultivating compassion [31].

CFT aims to support individuals in transitioning from a state of self-blame with disabling effects to a place where they can assume responsibility for navigating extraordinarily arduous and demanding circumstances. Developing self-compassion towards the challenges associated with bipolar disorder is a crucial aspect of treatment, which may involve addressing clients’ anger and sadness as they envision a life unaffected by BD. Additionally, CFT strives to foster an awareness of the strengths that can be associated with bipolar disorder [27].

Clients undergoing CFT training can develop an understanding that their engagement in self-attack is likely to yield effects on their minds and brains comparable to actual external attacks. With the permission of the relevant authorities, it is possible to share details concerning the augmented recurrence pattern observed in environments characterized by intense emotional expression. These environments are often linked to heightened stress levels resulting from frequent criticism and an emotional climate of hostility (136).

Asking clients to vocalize their aggressive thoughts about the difficulties and obstacles associated with bipolar disorder can prove beneficial in heightening their awareness of the destructive effects caused by chronic self-attack [27]. According to CFT, the attachment and caregiving systems have evolved as mechanisms that regulate psychophysiological, emotional, and behavioral aspects, forming the basis for overall well-being and health (Brown & Brown, 2015). Consequently, therapeutic approaches aim to shift individuals away from excessively defensive or competitive motivations, fostering their transformation into individuals characterized by compassion and empathy.Individuals diagnosed with bipolar disorder encounter various intricate obstacles on their path to recovery, such as managing mood fluctuations, grappling with the apprehension of relapse, and navigating potentially complicated states of ambivalence regarding manic episodes and the utilization of psychiatric medications.

The pursuit of high standards often poses a hindrance to the recovery process in individuals with bipolar disorder. However, attaining high-quality standards can create challenges within the recovery process. A self-destructive behavior pattern of self-attack can significantly impede one’s capacity to handle setbacks effectively [32].

In this context, Raniere et al. [33] conducted a study in 2006, revealing that performance-oriented people, characterized by stringent self-standards and self-criticism, may exhibit susceptibility to depressive symptoms. Moreover, when individuals with bipolar disorder encounter life events, their hypomanic tendencies become intertwined with this particular style. Conversely, the attachment-related personality style serves as a protective element against depression when individuals with bipolar disorder encounter mutually unfavorable circumstances. These findings align with the outcomes of the current study, providing further support [33]. Rosenfarb et al. conducted a separate research that yielded findings indicating higher levels of self-criticism and dependency among women experiencing depression with unipolar disorder as compared to the non-psychiatric control group. In an independent study conducted by Braehler et al., it was found that participants who underwent CFT demonstrated significant clinical advancements and a substantial enhancement in compassion. Moreover, they experienced a more significant decrease in depression levels and a reduction in feelings of social isolation. The participants who received the CFT exhibited minimal side effects, demonstrated a low participant attrition rate, and were perceived as highly acceptable. Throughout this study, a consistent decline in compassion was observed within the group undergoing focus-based therapy, displaying a noteworthy distinction from the control group. These outcomes align with the present study’s findings, further reinforcing their validity [34].

Gumley et al. conducted a study exploring the application of CFT in understanding specific issues related to psychosis. Their findings suggested that CFT holds promise as an effective intervention for individuals with psychotic symptoms, thus corroborating the results obtained in the present study [35]. The findings by Laithwaite et al. similarly demonstrated significant improvement across various scales, including social comparison, shame, depression, self-esteem, and general psychopathology.

These results align with the present study and the study conducted by Gumley et al. In a 2019 study led by Heriot-Maitland et al., it was found that targeted therapy exhibited effectiveness in enhancing the compassion index. The researchers recommended the application of CFT as a potential approach for the treatment of distressing auditory hallucinations [36]. The findings presented by Khalat Bari et al. indicated that implementing CFT yielded positive outcomes concerning body image and marital satisfaction among women diagnosed with breast cancer [37]. Toole et al. [38] conducted a study in which they observed that engaging in self-compassion meditation training led to a decrease in disturbances associated with body image and an improvement in self-compassion. To analyze the findings from research conducted in this domain and compare them with the outcomes of the current study, it can be inferred that the impact of CFT on treatment outcomes in individuals with bipolar disorder, particularly with variables such as shame, self-compassion, and self-criticism can be promising. Through various studies employing diverse methodologies and encompassing distinct target populations, it has been noted that targeted interventions hold the potential to be effective in addressing the needs of individuals with bipolar disorder and other mood disorders. The operational principle underlying focus-based therapy lies in its initial therapeutic approach, Compassionate Mind Cultivation (CMT), within the framework of CFT. CMT refers to strategies that commonly facilitate individuals in developing and experiencing various dimensions of compassion towards themselves and others. The primary objective of CMT is to foster improving motivation, empathy, sensitivity, and distress tolerance compassionately by employing targeted exercises. These exercises assist individuals in developing a mindset that is impartial and free from assigning blame or passing judgment. When individuals experience challenges related to self-attacking emotions, the therapist can assist in exploring the underlying motivations and potential origins of these self-directed attacks. Additionally, they can explore possible factors that contribute to individuals’ compliance or surrender to these self-attacks. This approach may entail envisioning the self-attack as a separate individual. Within this therapy, individuals are asked to describe the characteristics of that "person" and express their sentiments towards them, facilitating a more comprehensive comprehension of self-critical tendencies. The question design process can help individuals who struggle with experiencing and expressing compassion by identifying and addressing any barriers that may hinder their ability to express compassion. However, it can be said that a discernible mechanism of action and a cohesive therapeutic approach exist, necessitating further comprehensive investigations to establish the stability of this treatment method.

## Conclusion

The findings from the present study revealed a significant difference between the two groups, where the CFT and the standard treatment exhibited a significant difference when compared to the standard treatment alone. This disparity was observed across various variables, including shame, self-compassion, anxiety associated with bipolar disorder, and self-criticism. It seems that CFT exerts a significant effect on the treatment outcomes of individuals diagnosed with bipolar disorder.

## Conflict of Interest

None declared.

## References

[R1] (2011). Medscape..

[R2] Melisa S (USA:). Bipolar disorder. 1st ed.

[R3] Barekatin M, Tavakkoli M, Molavi H, Maroofi M, Salehi M (2007). (2007) Normality, reliability, and validity of Young Mania Rating
Scale. Journal of Psychology.

[R4] Diagnostic and (2013).

[R5] Merikangas KR, Akiskal HS, Angst J, Greenberg PE, Hirschfeld RM, Petukhova M, Kessler RC (2007). Lifetime and 12-month prevalence of bipolar spectrum disorder in
the
National Comorbidity Survey replication. Archives of general psychiatry.

[R6] Mohammadi MR, Davidian H, Noorbala A, Malekafzali H, Naghavi H, Pouretemad H, et al (2005). An epidemiological survey of psychiatric disorders in Iran. J Clin Pract & Mohammadi, M Epidem Ment Health.

[R7] Ball J, Mitchell P, Malhi G, Skillecorn A, Smith M (2003). Schema-focused cognitive therapy for bipolar disorder: reducing
vulnerability to relapse through attitudinal change. Australian & New Zealand Journal of Psychiatry.

[R8] Word Health (Available from http://apps.who.int/iris/bitstream/10665/57224/1/ bu0610
).

[R9] Revicki DA, Hirschfeld RM, Ahearn EP, Weisler RH, Palmer C, Keck Jr (2005). Effectiveness and medical costs of divalproex versus lithium in
the
treatment of bipolar disorder: results of a naturalistic clinical trial. Journal of affective disorders.

[R10] Gouveia J, Matos M (2011). Can shame memories become a key to identity The centrality of
shame
memories Pinto .predicts psychopathology. Applied Cognitive Psychology.

[R11] Gilbert P (2014).

[R12] Scott J, Mansell W (2005). The nature and treatment of depression in bipolar F -30 disorder:
A
review and implications for future psychological investigation. Clinical Psychology Review.

[R13] Jamison KR, Goodwin FK (1990). Manic–depressive illness. New York, NY: Oxford University.

[R14] Gilbert P, Clarke M, Hempel S, Miles JN, Irons C (2004). Criticizing and reassuring oneself: An exploration of forms,
.styles and
reasons in female students. British Journal of Clinical Psychology.

[R15] Straccamore F, Ruggi S, Lingiardi V, Zanardi R, Vecchi S, Oasi O (2017). Personality factors and depressive 37 configurations an
exploratory study
in an Italian clinical sample. Frontiers in psychology.

[R16] McMurrich SL, Johnson SL (2009). The role of depression, shame-proneness, and guilt-proneness in
predicting criticism of relatives towards people with bipolar disorder. Behavior therapy.

[R17] Scott J (2001). Cognitive therapy as an adjunct to medication in bipolar disorder. Br J Psychiatry.

[R18] Gitlin MJ, Swendsen J, Heller TL, Hammen C (1995). Relapse and impairment in bipolar disorder. American Journal of Psychiatry.

[R19] Oud M, Mayo-Wilson E, Braidwood R, Schulte P, Jones SH, Morriss R, Kupka R, Cuijpers P, Kendall T (2016). Psychological interventions for adults with bipolar disorder:
systematic
review and meta-analysis. The .British Journal of Psychiatry.

[R20] MacBeth A, Gumley A (2012). Exploring compassion: A meta - analysis of the association
between self -
compassion and psychopathology. Clinical psychology review.

[R21] Rosenfarb IS, Becker J, Khan A, Mintz J (1998). Dependency and self‐criticism in bipolar and unipolar depressed
women. British Journal of Clinical Psychology.

[R22] Gharraee B, Tajrishi KZ, Farani AR, Bolhari J, Farahani H (2018). A randomized controlled trial of compassion focused therapy for
social
anxiety disorder. Iranian Journal of Psychiatry and Behavioral Sciences.

[R23] Pascual-Sánchez A, Montes-Rodríguez JM, Jenaro-Río C, Saiz-Ruiz J (2019). Validation of a brief scale for the assessment of distress
associated to
bipolar disorder. The European Journal of Psychiatry.

[R24] Patel R, Reiss P, Shetty H, Broadbent M, Stewart R, McGuire P, et al (2015). Do antidepressants increase the risk of mania and bipolar
disorder in
people with depression A retrospective electronic case register cohort study. BMJ Open.

[R25] Kwentus J, Riesenberg RA, Marandi M, Manning RA, Allen MH, Fishman RS, et al (2012). Rapid acute treatment of agitation in patients with bipolar I
disorder: a
multicenter, randomized, placebo-controlled clinical trial with inhaled
loxapine. Bipolar Disord.

[R26] Bauer M, Alda M, Priller J, Young LT (2003). Implications of the neuroprotective effects of lithium for the
treatment
of bipolar and neurodegenerative disorders. Pharmacopsychiatry.

[R27] Jiang Y, You J, Zheng X, Lin MP (2017). The qualities of attachment with significant others and self -
compassion
protect adolescents from non suicida l self - injury. School psychology quarterly.

[R28] Neff KD, Dahm KA (2015). Self-compassion: What it is, what it does, and how it relates to
mindfulness. Handbook of mindfulness and self-regulation.

[R29] Tohen M, Goldberg JF, Arrillaga AM, Azorin JM, Vieta E, HardyBayle MC, et al (2003). A 12-week, double-blind comparison of olanzapine vs haloperidol
in the
treatment of acute mania. Arch Gen Psychiatry.

[R30] Tohen M, Baker RW, Altshuler LL, Zarate CA, Suppes T, Ketter TA, et al (2002). Olanzapine versus divalproex in the treatment of acute mania. Am J Psychiatry.

[R31] Neff KD, Hsieh YP, Dejitterat K (2005). Self compassion, achievement goals, and copi ng with academic
failure. Self and identity.

[R32] Lowens I (2010). Compassion focused therapy for people with bipolar disorder
International. Journal of Cognitive Therapy.

[R33] Raniere EL, Alloy LB, Abramson LY (2006). Depressive personality styles and bipolar spectrum
Francisdisorders:
Prospective tests of the event congruency hypothesis. Bipolar Disorders.

[R34] Braehler C, Gumley A, Harper J, Wallace S, Norrie J, Gilbert P (2013). Exploring change processes in compassion focused therapy in
psychosis:
Results of a feasibility randomized controlled trial. British Journal of Clinical Psychology.

[R35] Gumley A, Braehler C, Laithwaite H, MacBeth A, Gilbert P (2010). A compassion focused model of recovery after psychosis. International Journal of Cognitive Therapy.

[R36] Heriot-Maitland C, McCarthy-Jones S, Longden E, Gilbert P (2019). Compassion focused approaches to working with .distressing voices. Frontiers in psychology.

[R37] Khalatbari J, Hemmati SV, Mohammadi H (2018). Effect of Compassion-Focused Therapy on Body Image and Marital
Satisfaction in Women with Breast Cancer. IRANIAN QUARTERLY JOURNAL OF BREAST DISEASE.

[R38] Toole AM, Craighead LW (2016). Brief self-compassion meditation training for body image distress
in
young adult .women. Body image.

